# Performance assessment of VSC-based HVDC system in asynchronous grid interconnection: Offline and real-time validation of control design with symmetric optimum PI tuning

**DOI:** 10.1016/j.heliyon.2024.e35624

**Published:** 2024-08-02

**Authors:** Adesh Kumar Mishra, Saurabh Mani Tripathi, Omveer Singh, Ankit Kumar Srivastava, Thiyagarajan Venkatraman, Raghavendra Rajan Vijayaraghavan, Sachin Kumar, Rajvikram Madurai Elavarasan, Lucian Mihet-Popa

**Affiliations:** a Department of Electrical Engineering, Gautam Buddh University, Greater Noida, 201312, U.P, India; b Department of Electrical Engineering, Kamla Nehru Institute of Technology, Sultanpur, 228118, U.P, India; c Department of Electrical Engineering, Dr. Ramanohar Lohia Avdh University, Ayodhya, 224001, U.P, India; d Department of Electrical and Electronics Engg., Sri Sivasubramaniya Nadar College of Engineering, Kalavakkam, Chennai, 603110, TN, India; e Automotive Department, Harman Connected Services India Pvt. Ltd., Bengaluru, 560066, India; f Govind Ballabh Pant Institute of Engineering & Technology, Pauri Garhwal, 246194, India; g School of Engineering and Energy, Murdoch University, WA, 6168, Australia; h Faculty of Information Technology, Engineering and Economics, Østfold University College, Kobberslagerstredet 5, 1671, Fredrikstad, Norway

**Keywords:** Asynchronous grid interconnection, HVDC system, Offshore AC network, Onshore AC network, PI controllers, Real-time simulation, Symmetric optimum PI tuning, Voltage source converter (VSC)

## Abstract

Asynchronous interconnection is essential for integrating AC networks operating at different frequencies, typically 50 Hz and 60 Hz. This need arises from distributed power generation methods, including offshore renewable sources and diverse regional grid configurations. Advanced strategies are required to overcome these frequency differences and ensure uninterrupted power transfer.

High-Voltage Direct Current (HVDC) transmission systems facilitate efficient power exchange, enhancing grid reliability and stability. This study focuses on optimizing the Proportional-plus-Integral (PI) controller parameters within a 20 MVA Voltage Source Converters (VSC)-based HVDC system to enable asynchronous interconnection between offshore and onshore AC networks. The offshore VSC regulates active and reactive power, while the onshore VSC controls DC voltage and reactive power. A vector control approach with symmetric optimum PI tuning is proposed for a comprehensive performance assessment of the VSC-based HVDC transmission system.

The effectiveness of the tuned PI controller parameters is evaluated through four test cases using MATLAB/Simulink for offline simulation and Typhoon HIL604 for real-time validation. These cases involve abrupt changes in reference active and reactive power for the offshore VSC; and in reference reactive power and DC voltage for the onshore VSC. Results demonstrate rapid and satisfactory dynamic performance across all test cases, as evidenced by offline simulations and real-time validation.

The validation highlights the effectiveness of the proposed control design with symmetric optimum PI tuning, confirming its ability to enhance the overall performance of the HVDC transmission system for efficient asynchronous interconnection.

**Table:** 

**Nomenclature**	
***List of Abbreviations***	
AC	Alternating current
CSC	Current source converter
DC	Direct current
EMTDC	Electromagnetic transients including DC
HIL	Hardware-in-the-loop
HVDC	High voltage direct current
MATLAB	Matrix laboratory
MW	Megawatt
MVAR	Megavolt-ampere-reactive
PERC	Power and energy research centre
PI	Proportional-plus-integral
PLL	Phase-locked-loop
PSCAD	Power systems computer aided design
RMS	Root-mean-square
SFC	Supplementary frequency control
SGEC	Synchronous generator emulation control
VSC	Voltage source converter
*List of Symbols*	
a	Parameter representing a balance between closed-loop pole damping and dynamic responses
*C*_OFF_	DC link capacitors at the offshore VSC terminal (μF)
*C*_ON_	DC link capacitors at the onshore VSC terminal (μF)
$e_{dOFF}$	*d*-axis component of offshore AC voltage (*V*)
$e_{qOFF}$	*q*-axis component of offshore AC voltage (*V*)
$e_{dON}$	*d*-axis component of onshore AC voltage (*V*)
$e_{qON}$	*q*-axis component of onshore AC voltage (*V*)
$G_{1}$, $G_{2}$	Benchmark transfer functions
$G_{OL\_IN}$	Open-loop transfer function for the inner current control loops
$G_{OL\_dcON}$	Open-loop transfer function for the outer DC voltage control loop in the onshore control system
$i_{d}^{*}$	Reference value of *d*-axis component of AC current (*A*)
$i_{q}^{*}$	Reference value of *q*-axis component of AC current (*A*)
$i_{dOFF}$	*d*-axis component of offshore AC current (*A*)
$i_{qOFF}$	*q*-axis component of offshore AC current (*A*)
$i_{dOFF}^{*}$	Reference value of *d*-axis component of offshore AC current (*A*)
$i_{qOFF}^{*}$	Reference value of *q*-axis component of offshore AC current (*A*)
$i_{dON}$	*d*-axis component of onshore AC current (*A*)
$i_{qON}$	*q*-axis component of onshore AC current (*A*)
$i_{dON}^{*}$	Reference value of *d*-axis component of onshore AC current (*A*)
$i_{qON}^{*}$	Reference value of *q*-axis component of onshore AC current (*A*)
$I_{dcON}$	DC current (*A*)
$K_{g}$	Plant gain
$K_{1}$	Proportional gain of a typical PI controller
$K_{p}$	Proportional gain of the inner PI controller
$K_{pc}$	Proportional gain of the inner PI controller employed within the offshore control system
$K_{pi}$	Proportional gain of the inner PI controller employed within the onshore control system
$K_{pv}$	Proportional gain of the outer DC voltage PI controller employed within the onshore control system
$L_{g}$	Coupling reactor at onshore VSC terminal (*mH*)
$L_{s}$	Coupling reactor at offshore VSC terminal (*mH*)
$P_{OFF}$	Instantaneous active power supplied by offshore AC network (MW)
$P_{OFF}^{*}$	Reference value of instantaneous active power supplied by offshore AC network (MW)
$P_{ON}$	Instantaneous active power delivered to onshore AC network (MW)
$Q_{OFF}$	Instantaneous reactive power injected into/absorbed from offshore AC network (MVAR)
$Q_{OFF}^{*}$	Reference value of instantaneous reactive power injected into/absorbed from offshore AC network (MVAR)
$Q_{ON}$	Instantaneous reactive power injected into/absorbed from onshore AC network (MVAR)
$Q_{ON}^{*}$	Reference value of instantaneous reactive power injected into/absorbed from onshore AC network (MVAR)
$R_{g}$	Resistance of the coupling reactor at onshore VSC terminal (Ω)
$R_{s}$	Resistance of the coupling reactor at offshore VSC terminal (Ω)
$T_{c}$	Sampling time for the inner loop (μs)
$T_{d}$	Dominant time constant of the plant (*s*)
$T_{1}$	Integral-time constant of a typical PI controller (*s*)
$T_{i}$	Integral-time constant of the inner PI controller (*s*)
$T_{ic}$	Integral-time constant of the inner PI controller employed within the offshore control system (*s*)
$T_{ii}$	Integral-time constant of the inner PI controller employed within the onshore control system (*s*)
$T_{iv}$	Integral-time constant of outer DC voltage PI controller employed within the onshore control system (*s*)
$T_{m}$	Minor time constant of the plant (*s*)
$T_{v}$	Sampling time for outer DC voltage control loop (μs)
$v_{d}^{*}$	Reference value of *d*-axis component of VSC voltage (*V*)
$v_{q}^{*}$	Reference value of *q*-axis component of VSC voltage (*V*)
$v_{dOFF}^{*}$	Reference value of *d*-axis component of offshore VSC voltage (*V*)
$v_{qOFF}^{*}$	Reference value of *q*-axis component of offshore VSC voltage (*V*)
$v_{dON}^{*}$	Reference value of *d*-axis component of onshore VSC voltage (*V*)
$v_{qON}^{*}$	Reference value of *q*-axis component of onshore VSC voltage (*V*)
$V_{dcON}$	DC voltage (*V*)
ζ	Damping ratio
$\theta_{s}$	Phase angle of offshore AC network (*rad*)
$\theta_{g}$	Phase angle of onshore AC network (*rad*)
$\omega_{s}$	Angular frequency of offshore AC network (*rad/s*)
$\omega_{g}$	Angular frequency of onshore AC network (*rad/s*)

## 1 Introduction

Electricity is crucial for national development, but sustainable growth challenges necessitate adopting renewable power sources to mitigate environmental impact and reduce fossil fuel depletion, essential for rising global electricity demand [[1], [2], [3], [4], [5]]. As the demand for renewable power sources grows, distributed power generation from offshore renewable installations like wind farms, solar photovoltaic and tidal energy sites has become increasingly prevalent [2,5]. These sources inject power into grids with varying configurations and operational frequencies across different regions. Asynchronous interconnection refers to the ability to connect AC networks operating at different frequencies [6,7]. This necessity arises due to the increasingly widespread adoption of distributed power generation methods, particularly offshore renewable power sources [7] like wind farms and tidal energy installations. These sources often feed power into grids with different configurations and operating frequencies across various regions.

High Voltage Direct Current (HVDC) systems operate independently of frequency, making them ideal for interconnecting grids with differing frequencies. HVDC systems enable the smooth integration of AC networks with differing frequencies through the conversion of AC power to DC for transmission and its subsequent reconversion back to AC at the receiving end [6,8]. This capability not only enables the seamless integration of renewable power sources but also enhances overall grid reliability and stability [9]. By enabling seamless power transfer despite frequency mismatches, HVDC systems play a pivotal role in modernizing power infrastructure and supporting sustainable energy transitions.

The Voltage Source Converter (VSC)-based HVDC transmission system has emerged as a forefront technology, renowned for its cost-effectiveness and reliability in facilitating asynchronous interconnection between two AC networks [[6], [7], [8]]. This technological advancement represents a key milestone in power transmission engineering, addressing the efficient transfer of power between AC networks operating at different frequencies [9,10]. In this system configuration, two VSCs interconnected via an HVDC cable enable precise control and management of power flow, ensuring seamless integration and compatibility between interconnected grids [7,11]. The benefits of VSC-based HVDC systems over conventional counterparts are manifold, including rapid and independent control of active and reactive power, leading to enhanced system flexibility and efficiency. Moreover, it contributes to improved power quality by minimizing voltage fluctuations and harmonics in transmission, thereby enhancing transmission stability and grid reliability [7,[11], [12], [13], [14], [15]]. Additionally, the utilization of VSC technology reduces short-circuit currents, thereby enhancing overall system safety and longevity, while requiring fewer harmonic filters, consequently reducing equipment costs, and simplifying system design. Furthermore, its compatibility with renewable power sources enhances its appeal, enabling seamless integration with solar, wind, and other sustainable energy systems [[16], [17], [18], [19]]. An extensive exploration of HVDC technology, as detailed in Ref. [17], includes an analysis of its historical development and current status, alongside an examination of its notable characteristics, constraints, and practical applications. Additionally, the authors in Ref. [19] provide an overview, covering VSCs, Current-Source Converters (CSCs), converter substation components, control strategies, implementation challenges, and prospects within HVDC applications. Studies such as the one by Ufa et al. [8] have implemented a VSC-based HVDC model employing a hybrid simulation concept using HRTSim and MATLAB/Simulink to verify both the static and dynamic performances of the hybrid model of VSC-HVDC. This approach provides a robust framework for analyzing the effectiveness of VSC-HVDC systems in maintaining grid stability and reliability under various operating conditions.

Within the domain of VSC-based HVDC transmission systems, the design of control systems emerges as a crucial area of focus [20]. The authors in Ref. [9] introduce a dual-loop supplementary frequency control scheme designed for back-to-back VSC-based HVDC systems connecting asynchronous AC grids. Their study focused on enhancing both transient and steady-state characteristics of the system's frequency response, demonstrating the effectiveness of the control strategy in optimizing HVDC system performance. Comprehensive overviews provided by Refs. [21,22] highlight crucial multi-level converter topologies, control strategies, and considerations regarding reliability and protection. The discussion in Ref. [8] revolves around the outcomes of implementing the VSC-based HVDC model using a hybrid simulation method for electric power systems. Additionally, studies such as [23] contribute significantly to power system stability by introducing dynamic models tailored for VSC-HVDC, emphasizing filter design and Phase-Locked-Loop (PLL) implementation. The convolution-based approach, along with adaptive sliding mode control incorporating time delay, as presented in Ref. [24], is employed to enhance power flow reference tracking in VSC-based HVDC transmission systems. This approach is specifically designed to ensure robust behavior and achieve chatter-free control, highlighting significant advancements in control methodologies aimed at enhancing the stability and performance of HVDC systems. Chen et al. [25] introduced a reactive power suppression approach using frequency feedback, employing PI controllers in MATLAB/Simulink for VSC-based HVDC systems. Their research specifically aimed to suppress low-frequency grid oscillations while ensuring minimal impact on transmitted power. Wang et al. [26] introduced an adaptive strategy employing linear active disturbance rejection control with PI controllers in RT-LAB and MATLAB/Simulink for VSC-based HVDC systems, focusing on improving the response speed and robustness of DC voltage regulation. The authors in Ref. [27] developed a frequency control approach integrating PI and Fuzzy Logic Controller in PSCAD/EMTDC, aimed at significantly enhancing frequency stability in power systems. The authors in Ref. [10] introduces a communication-free frequency regulation scheme for interconnected grids with VSC-HVDC links, employing Synchronous Generator Emulation Control to balance grid frequencies through adaptive adjustment of VSC converter powers, verified via simulations in PSCAD/EMTDC.

Furthermore, innovative control strategies, such as the direct-current vector control discussed in Ref. [13], offer compelling alternatives to conventional methods, prompting comparative analyses for performance evaluation. The research [28] focuses on controlling VSC-HVDC systems connecting two power grids via a long cable using a back-stepping design approach, ensuring precise voltage regulation, power factor correction, and active power control at the inverter station, supported by a high-gain observer for state estimation and stability analysis. The authors in Ref. [29] provides a comprehensive overview of recent advancements in VSC-based HVDC technology, including selected key multilevel converter topologies, discussions on control and modeling methods, and a compilation of VSC-based HVDC installations worldwide. The authors in Ref. [20] deliver a thorough examination of state-of-the-art control methodologies designed to maximize the efficiency of VSC-based HVDC systems.

In addition to designing the control system, making the right choices for controller parameters significantly contributes to improving the stability of the VSC-based HVDC system [20,28]. A tabulated summary of several controllers is provided in Ref. [30], effectively highlighting their distinctive advantages and drawbacks.

Achieving optimal performance requires precise controller parameter adjustments, posing a challenging task for control systems designers [26,[31], [32], [33]]. Table 1 presents a brief overview of research conducted in recent years on VSC-based HVDC systems, offering insights into the methodologies employed and key outcomes achieved. Building upon this, the following elucidates key points that clarify the existing research gap:

1. PI controllers are widely favored in VSC-based HVDC transmission systems for their role in achieving stability and control performance, yet their precise tuning is frequently overlooked, presenting challenges for designers.
2. There is limited exploration of PI controller optimization that simultaneously enhances closed-loop performance and system robustness in VSC-based HVDC systems.
3. Effective strategies are needed to address the specific challenges of enhancing stability and dynamic response in asynchronous AC network interconnections through optimized PI controllers within VSC-based HVDC systems.

**Table 1:** Review of VSC-based HVDC systems: A brief research overview.

Sr.	Year	Author	Methodology/Approach	Controller	Tools	Outcomes	Ref.
1.	2016	Guan et al.	Synchronous generator emulation control (SGEC)	PI	PSCAD/EMTDC	Balances grid frequencies through adaptive adjustment of VSC powers.	[10]
2.	2017	Hamache et al.	Adaptive sliding mode with time delay, employing a convolution-based Approach	PI/Sliding mode control	Not Specified	Ensuring robust behavior and chatter-free control.	[24]
3.	2019	Ufa et al.	Implementation of a VSC-based HVDC model employing a hybrid simulation concept	Not Specified	HRTSim and MATLAB/Simulink	Verification of the static and dynamic performances of the hybrid model of VSC-HVDC.	[8]
4.	2019	Zeng et al.	Dual-loop supplementary frequency control (SFC) strategy	PI	MATLAB/Simulink	Enhancing the transient and steady-state characteristics of the system's frequency response.	[9]
5.	2022	Chen et al.	Reactive power suppression approach utilizing frequency feedback.	PI	MATLAB/Simulink	Suppression of low-frequency oscillations in the grid effectively without impacting the transmitted power.	[25]
6.	2023	El Myasse et al.	A back-stepping design technique to develop a nonlinear controller for VSC-HVDC systems, focusing on voltage regulation, power factor correction, and active power control, complemented by a high-gain observer for state estimation.	Nonlinear controller	MATLAB/Simulink	Implemented nonlinear controller, accurate state estimation, validated stability through simulations.	[28]
7.	2023	Wang et al.	An adaptive strategy employing linear active disturbance rejection control	PI	RT-LAB and MATLAB/Simulink	Effectively improving the response speed and robustness of the DC voltage.	[26]
8.	2024	Xing et al.	A frequency control approach utilizing fuzzy logic control	PI/Fuzzy Logic Controller	PSCAD/EMTDC	Significantly enhancing frequency stability.	[27]
9.	2024	Adesh et al.	A vector control strategy employing symmetric optimum PI tuning	PI	MATLAB/Simulink	Stable and efficient power transfer without encountering severe transient fluctuations.	This paper

In formal terms, the problem statement can be formulated as follows:

“Tune the parameters of the PI controllers optimally within the control systems of a VSC-based HVDC system to enhance closed-loop control performance while maintaining the robustness margin, ensuring seamless power transmission between AC networks operating at different frequencies.”

Furthermore, following are the points that underscore the contributions of this research study:

1. This study offers a systematic approach to tuning PI controllers using the symmetric optimum technique for a 20 MVA VSC-based HVDC transmission system. Unlike previous studies, this work optimizes both closed-loop control performance and system robustness, addressing a critical challenge often overlooked by designers.
2. The study demonstrates that well-tuned PI controllers are crucial for optimal closed-loop control performance and system stability. The proposed technique significantly enhances system stability and dynamic response in asynchronous AC network interconnections, ensuring robust control across various operational environments.
3. To ensure practical applicability and reliability, the control strategy is validated through both offline simulations using MATLAB/Simulink and real-time testing with Typhoon HIL604. This dual validation confirms that the control strategy performs reliably under real-world conditions, strengthening the credibility of the research.
4. The comprehensive control methodology integrates finely tuned PI controller parameters, leading to substantial performance improvements. This work significantly contributes to the field of VSC-based HVDC systems by addressing the need for precise controller adjustments.

The paper is organized in the following manner: Section 2 presents a comprehensive block-diagram representation of the proposed VSC-based HVDC transmission system. In Section 3, the control strategy adopted and the estimation of current references for the control system of the proposed VSC-based HVDC transmission system are introduced. Section 4 elaborates on the symmetric optimum tuning technique employed for fine-tuning of the PI controller parameters. Moving forward to Section 5, the paper investigates the dynamic performance of the VSC-based HVDC transmission system across four test cases, providing detailed simulation results. In Section 6, the real-time experimental validation of dynamic performance is carried out. Finally, Section 7 presents the conclusions drawn from the study.

## 2 VSC-based HVDC transmission system

**Fig. 1:**
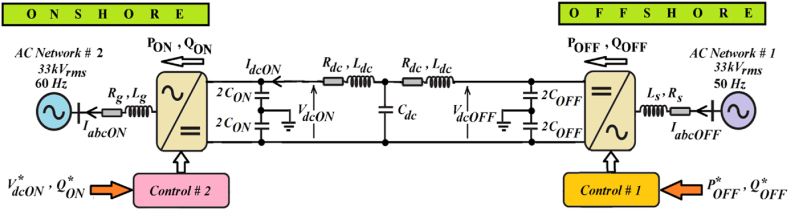
Proposed VSC-based HVDC transmission system.

Fig. 1 provides a comprehensive block-diagram of the VSC-based HVDC transmission system proposed for facilitating the interconnection between offshore and onshore AC networks operating at distinct frequencies, namely 50 Hz and 60 Hz, respectively. The primary goal of this system is to efficiently transfer power from the offshore AC network to the onshore AC network. The proposed VSC-based HVDC transmission system comprises two VSCs: one located at the offshore and the other at the onshore, interconnected via a DC cable. Moreover, *C*_ON_ represents the DC link capacitors at the onshore VSC terminal, while *C*_OFF_ represents the DC link capacitors at the offshore VSC terminal. Each VSC operates independently, enhancing system flexibility and reliability.

**Fig. 2:**
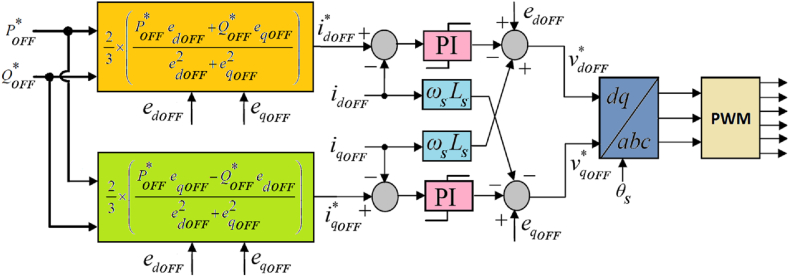
Control # 1 for offshore VSC.

**Fig. 3:**
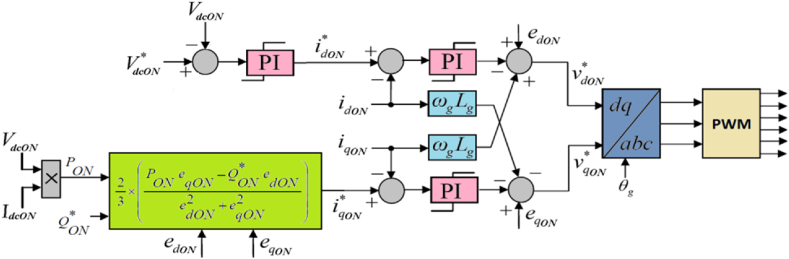
Control # 2 for onshore VSC.

Furthermore, the control systems for both the offshore (Control #1) and onshore (Control #2) VSCs are elaborated in Fig. 2, Fig. 3, respectively. These control systems are based on vector control principle and are implemented within the synchronous rotating *d-q* reference frame. The offshore control system oversees the regulation of both active and reactive powers, whereas onshore control system focuses on regulating DC voltage and reactive power to uphold system stability.

To ensure seamless operation, the angular frequencies ($\omega_{s}$ and $\omega_{g}$) and phase angles ($\theta_{s}$ and $\theta_{g}$) of the offshore and onshore AC networks, respectively, which are critical for the operation of both offshore and onshore VSC control systems, are precisely determined using the PLL technique. This technique guarantees the synchronization and alignment of frequencies and phase angles with their respective AC networks [34], ensuring optimal performance and seamless power transfer. For a comprehensive understanding, detailed parameters of the VSC-based HVDC transmission system are provided in the Appendix, enabling thorough analysis and evaluation of its operational characteristics.

## 3 Control strategy and estimation of reference currents

The instantaneous active power $P_{OFF}$ supplied by the offshore AC network and the instantaneous reactive power $Q_{OFF}$ injected into or absorbed from the offshore AC network are represented using *d-q* axes components [25,35] in the following manner:

(1) $$P_{OFF}=\frac{3}{2}\left(e_{dOFF}i_{dOFF}+e_{qOFF}i_{qOFF}\right)$$

(2) $$Q_{OFF}=\frac{3}{2}\left(e_{qOFF}i_{dOFF}-e_{dOFF}i_{qOFF}\right)$$

where $e_{dOFF}$ and $e_{qOFF}$ are the *d-q* axes components of the offshore AC voltage, respectively, and $i_{dOFF}$ and $i_{qOFF}$ are the *d-q* axes components of the offshore AC current, respectively. For control purposes, with reference to equations (1), (2), the determination of the *d-q* axes current references ($i_{dOFF}^{*}$ and $i_{qOFF}^{*}$) in the inner loop of the offshore control system relies on utilizing reference values for active and reactive powers ($P_{OFF}^{*}$ and $Q_{OFF}^{*}$), alongside *d-q* axes voltages ($e_{dOFF}$ and $e_{qOFF}$) measured at the offshore terminal [24]. Therefore,

(3) $$i_{dOFF}^{*}=\frac{2}{3}\times \left(\frac{P_{OFF}^{*}\;e_{dOFF}+Q_{OFF}^{*}\;e_{qOFF}}{e_{dOFF}^{2}+e_{qOFF}^{2}}\right)$$

(4) $$i_{qOFF}^{*}=\frac{2}{3}\times \left(\frac{P_{OFF}^{*}\;e_{qOFF}-Q_{OFF}^{*}\;e_{dOFF}}{e_{dOFF}^{2}+e_{qOFF}^{2}}\right)$$

Similarly, the instantaneous active power $P_{ON}$ delivered to the onshore AC network and the instantaneous reactive power $Q_{ON}$ injected into or absorbed from the onshore AC network are represented using *d-q* axes components [25,35] in the following manner:

(5) $$P_{ON}=\frac{3}{2}\left(e_{dON}i_{dON}+e_{qON}i_{qON}\right)$$

(6) $$Q_{ON}=\frac{3}{2}\left(e_{qON}i_{dON}-e_{dON}i_{qON}\right)$$

where $e_{dON}$ and $e_{qON}$ are the *d-q* axes components of the onshore AC voltage, respectively and $i_{dON}$ and $i_{qON}$ are the *d-q* axes components of the onshore AC current, respectively.

The determination of the *d*-axis current reference ($i_{dON}^{*}$) for the inner loop in the onshore control system is facilitated through an outer DC voltage PI controller. Meanwhile, in consideration of equations (5), (6), the computation of the *q*-axis current reference ($i_{qON}^{*}$) entails utilizing various parameters. These include the DC voltage ($V_{dcON}$), DC current ($I_{dcON}$), the reference value of reactive power ($Q_{ON}^{*}$), and the measured *d-q* axes voltages ($e_{dON}$ and $e_{qON}$) at the onshore VSC terminal [23,24]. This comprehensive approach ensures precise control over both active and reactive power flows.

(7) $$i_{dON}^{*}=K_{pv}\left(\frac{1+pT_{iv}}{pT_{iv}}\right)\left(V_{dcON}^{*}-V_{dcON}\right)$$

(8) $$i_{qON}^{*}=\frac{2}{3}\times \left(\frac{P_{ON}\;e_{qON}-Q_{ON}^{*}\;e_{dON}}{e_{dON}^{2}+e_{qON}^{2}}\right)$$

where $P_{ON}=V_{dcON}\times I_{dcON}$ (9)

Furthermore, PI controllers have been used within the inner control loops for both offshore and onshore VSCs to control respective *d-q* axes current components. Therefore,

(10) $$v_{dOFF}^{*}=K_{pc}\left(\frac{1+pT_{ic}}{pT_{ic}}\right)\left(i_{dOFF}^{*}-i_{dOFF}\right)$$

(11) $$v_{qOFF}^{*}=K_{pc}\left(\frac{1+pT_{ic}}{pT_{ic}}\right)\left(i_{qOFF}^{*}-i_{qOFF}\right)$$

where $v_{dOFF}^{*}$ and $v_{qOFF}^{*}$ denote the *d-q* axes reference offshore VSC voltages, while $K_{pc}$ and $T_{ic}$ represent the proportional gain and the integral time-constant, respectively, of the inner PI controllers employed within the offshore control system. Similarly,

(12) $$v_{dON}^{*}=K_{pi}\left(\frac{1+pT_{ii}}{pT_{ii}}\right)\left(i_{dON}^{*}-i_{dON}\right)$$

(13) $$v_{qON}^{*}=K_{pi}\left(\frac{1+pT_{ii}}{pT_{ii}}\right)\left(i_{qON}^{*}-i_{qON}\right)$$

where $v_{dON}^{*}$ and $v_{qON}^{*}$ denote the *d-q* axes reference onshore VSC voltages, while $K_{pi}$ and $T_{ii}$ represent the proportional gain and the integral time-constant, respectively, of the inner PI controllers employed within the onshore control system.

It is pertinent to highlight the deliberate choice of identical coupling reactors ($L_{s}$ = $L_{g}$ and $R_{s}$ = $R_{g}$) for both offshore and onshore VSCs, particularly considering their matching power ratings, operating voltages, and switching frequencies. Additionally, the sampling time ($T_{c}$) and switching frequency for the inner loop in both offshore and onshore control systems have been set at 100 μs and 5 kHz, respectively. This uniformity indicates that the inner control loops operate in the same way across both control systems. Thus, for simplicity and clarity, equations (10), (11), (12), (13) governing these inner current loops are deliberately represented identically, without distinguishing between onshore or offshore, as follows:

(14) $$v_{d}^{*}=K_{pg}\left(\frac{1+pT_{ig}}{pT_{ig}}\right)\left(i_{d}^{*}-i_{d}\right)$$

(15) $$v_{q}^{*}=K_{pg}\left(\frac{1+pT_{ig}}{pT_{ig}}\right)\left(i_{q}^{*}-i_{q}\right)$$

where $K_{p}=K_{pc}\;\left(=K_{pi}\right)$ and $T_{i}=T_{ic}\;\left(=T_{ii}\right)$ denote the proportional gain and integral time-constant, respectively, of the inner PI controllers. This ensures clarity in the representation of control strategies.

## 4 Tuning of the PI controllers

**Fig. 4:**
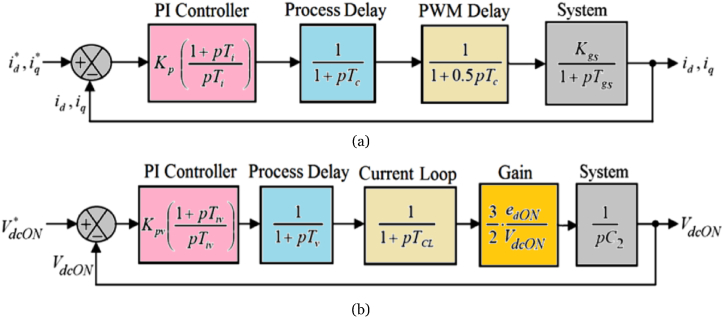
The mathematical block diagrams of (a) inner current control loop (b) onshore DC voltage control loop.

The open-loop transfer function for the inner current control loops, depicted in Fig. 4(a), can be written as:

(16) $$G_{OL\_IN}=K_{p}\left(\frac{1+pT_{i}}{pT_{i}}\right)\left(\frac{1}{1+pT_{c}}\right)\left(\frac{1}{1+o.5pT_{c}}\right)\left(\frac{K_{gs}}{1+pT_{gs}}\right)$$

where

(17) $$K_{gs}=\frac{1}{R_{s}}\left(=\frac{1}{R_{g}}\right)$$

(18) $$T_{gs}=\frac{L_{s}}{R_{s}}\left(=\frac{L_{g}}{R_{g}}\right)$$

Conversely, the outer DC voltage control loop in the onshore control system is illustrated in Fig. 4(b). The open-loop transfer function for the outer DC voltage control loop in the onshore control system can be expressed as follows:

(19) $$G_{OL\_dcON}=K_{pv}\left(\frac{1+pT_{iv}}{pT_{iv}}\right)\left(\frac{1}{1+pT_{v}}\right)\left(\frac{1}{1+pT_{CL}}\right)\left(\frac{3}{2}\times \frac{e_{dON}}{V_{dcON}}\right)\left(\frac{1}{pC_{ON}}\right)$$

where

(20) $$T_{CL}=a^{2}\times 1.5T_{c}$$

Moreover, $T_{v}$ represents the sampling time for the outer DC voltage control loop, while $K_{pv}$ and $T_{iv}$ denote the parameters of the PI controller within the DC voltage loop in the onshore control system.

To ensure optimal control performance and robustness in the VSC-based HVDC transmission system, parametric tuning of the PI controller is essential [20,31]. One widely employed technique for this purpose is the symmetric optimum, which is particularly effective when some system parameters are well-known. This method devises an optimal linear control system within the frequency domain. When a pole of the open-loop transfer function is close to or at the origin, the symmetric optimum technique becomes response across a broad frequency spectrum while simultaneously maximizing the phase margin to enhance the system's ability to tolerate delays [36]. When the plant model illustrated in the mathematical block diagram of the control loop closely aligns with the benchmark transfer function of the first form given by equation (21)

(21) $$G_{1}=\frac{K_{g}}{\left(1+pT_{d}\right)\left(1+pT_{m}\right)}\;;\;T_{d}\gg T_{m}$$

where $K_{g}$ represents the plant gain, $T_{d}$ represents the dominant time constant of the plant, and $T_{m}$ indicates the minor time constant of the plant; the symmetric optimum technique reliably provides the PI controller parameters as follows:

(22) $$K_{1}=\frac{T_{d}}{a\times T_{m}\times K_{g}}$$

(23) $$T_{1}=a^{2}\times T_{m}$$

On the other hand, when the plant model illustrated in the mathematical block diagram of the control loop closely aligns with the benchmark transfer function of the second form given by equation (24)

(24) $$G_{2}=\frac{K_{g}}{p\;\left(1+pT_{m}\right)}$$

The symmetric optimum technique reliably provides the PI controller parameters as follows:

(25) $$K_{1}=\frac{1}{a\times T_{m}\times K_{g}}$$

(26) $$T_{1}=a^{2}\times T_{m}$$

The parameter a, representing a balance between closed-loop pole damping and dynamic responses, can be chosen to achieve the desired damping ratio ζ and closed-loop control performance according to the following equation [31]:

(27) a=1+2ζ

In this study, plant models represented within the mathematical block diagrams of various control loops are simplified to closely align with a benchmark transfer function. This alignment facilitates the application of the symmetric optimum method for tuning their respective PI controllers. Employing the symmetric optimum technique with a value of a=2.4142, the PI controller parameters within the inner current control loops can be calculated utilizing equations (22), (23) as follows:

(28) $$K_{p}=\frac{T_{gs}}{a\times \left(1.5T_{c}\right)\times K_{gs}}=187.7779$$

(29) $$T_{i}=a^{2}\times 1.5T_{c}=8.7425\times 10^{-4}\;s$$

In contrast, employing the symmetric optimum technique with a=2.4142, the PI controller parameters within the outer DC voltage loop of the onshore control system can be established utilizing equations (25), (26) as follows:

(30) $$K_{pv}=\frac{2\times C_{ON}\times V_{dcON}}{3\times a\times e_{dON}\times \left(T_{v}+T_{CL}\right)}=0.2488$$

(31) $$T_{iv}=a^{2}\times \left(T_{v}+T_{CL}\right)=0.0109\;s$$

**Fig. 5:**
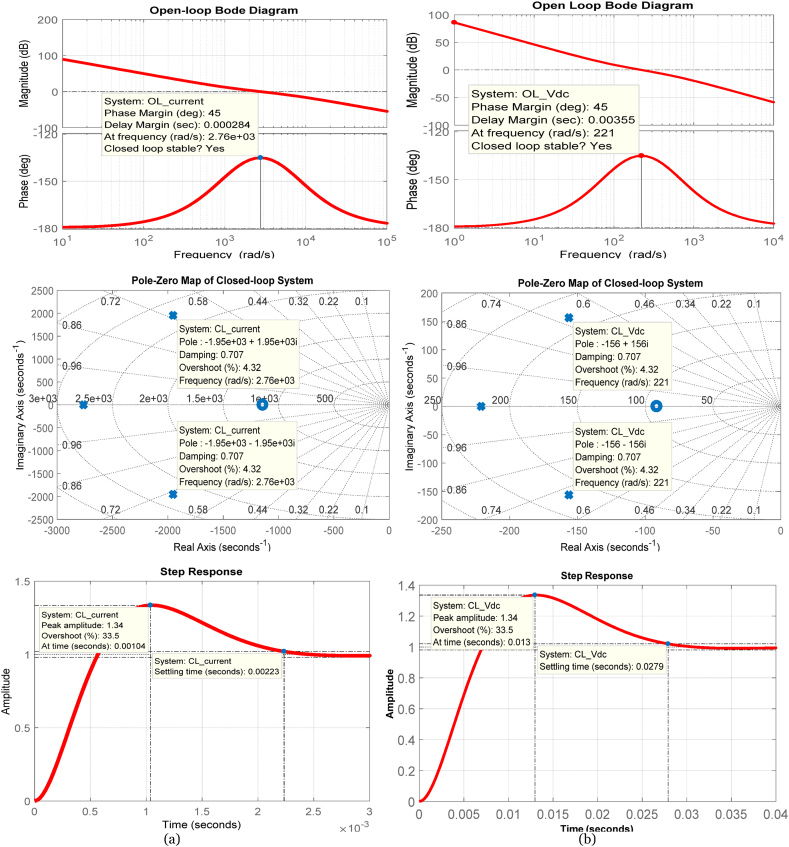
The open-loop Bode plots as well as pole-zero map and step response of closed-loop system with tuned parameters of PI controller employed in––(a) inner current loops (b) onshore DC voltage loop.

The results presented in Fig. 5 offer a comprehensive insight into the control performance of the system. Open-loop Bode plots illustrate the frequency response, while the pole-zero map clearly indicates stability with all poles situated in the left-half plane. Moreover, the step response analysis offers valuable insights into the system's dynamic behavior over time. A $45^{0}$ phase margin signifies stability and robustness, highlighting the reliability of the system. Furthermore, a damping factor of 0.707 for complex conjugate poles affirms stable behavior without oscillations, thereby confirming the reliability of the closed-loop control system. Collectively, these findings affirm the stability and reliability of the closed-loop control systems. Together, these findings underscore the effectiveness of the symmetric optimum technique for PI controller tuning in VSC-based HVDC system. However, addressing inherent limitations is crucial for a balanced perspective, as detailed below:

1. The symmetric optimum tuning of PI controller parameters assumes a linear model characterized by the transfer function, which simplifies the approach but may moderately limit its capability to comprehensively address the nonlinear dynamics often present in real-world applications.
2. The symmetric optimum method is particularly effective when specific system parameters are well-known, and the plant model closely aligns with predefined benchmark transfer functions. Nonetheless, its reliability can vary depending on factors such as plant gain, dominant time constant, and minor time constants, directly influencing its ability to optimize PI controller settings.
3. Furthermore, considerations related to variations in system parameters and operating conditions are critical as they can impact the performance and applicability of the technique. These variations may affect the stability and robustness of the control system, particularly when the actual plant characteristics severely deviate from the linear models employed in this study.

## 5 Offline simulation procedure and results

The test system involves a VSC-based HVDC transmission system designed for transferring power from an offshore AC network, operating at a frequency of 50 Hz, to an onshore AC network operating at a frequency of 60 Hz. A detailed listing of the system's parameters can be referred to in the Appendix. The system utilizes PI controllers to regulate various electrical parameters. The PI controllers ensure smooth control operation and promptly adjust to the new reference setting, ensuring stable and reliable power transfer. While analyzing the system's dynamic response reveals significant insights into the effectiveness of PI controllers in regulating both reactive and active power. To assess the efficacy of the PI controllers used, the offline simulation conducted with MATLAB/Simulink considers the following specific cases:

• **Case 1:** An abrupt change in reference active power for the offshore VSC from 10 MW to 16 MW at 0.6 s is considered. It analyses dynamic variations in active power transferred from the offshore network. The change is more than 50 % and an extremely adverse condition for the system.
• **Case 2:** The reference reactive power for the offshore VSC abruptly changes from +4 MVAR to −8 MVAR at 1.2 s. The change allows for comprehensive testing of the system's capability to manage reactive power. The capability is crucial for maintaining voltage stability across the offshore AC network. This change in reactive power indicates that initially, reactive power is being absorbed and then suddenly injected into the offshore AC network.
• **Case 3:** The reference reactive power for the onshore VSC abruptly changes from 0 MVAR to −5 MVAR at 1.8 s. It allows for evaluating the system's capability to manage reactive power within the onshore AC network when reactive power starts injecting into the onshore AC network. The analysis is essential for maintaining stable voltage levels and ensuring consistent delivery of high-quality power to the onshore AC network.
• **Case 4:** Lastly, an abrupt change is implemented in the reference DC voltage for the onshore VSC from 70 kV to 67 kV at 2.4 s. It indicates variations in the DC voltage level due to power flow fluctuations or AC network conditions. The objective is to assess the system's capability of stabilizing the DC voltage, ensuring consistent power transfer from offshore to onshore AC network.

### 5.1 Case 1

**Fig. 6:**
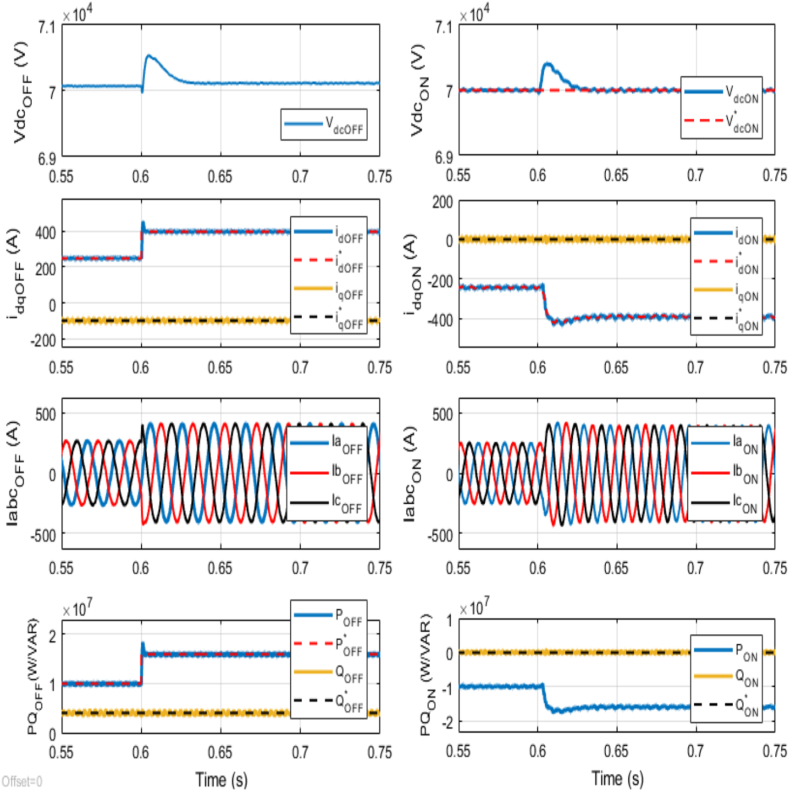
Dynamic performance of the VSC-based HVDC transmission system under abrupt change in Reference Active Power for Offshore VSC from 10 MW to 16 MW at time t = 0.6 s.

At the outset, the reference active and reactive powers for the offshore VSC are set at 10 MW and +4 MVAR, respectively. Initially, the offshore AC network supplies 10 MW of active power. However, at 0.6 s, the reference active power is abruptly increased to 16 MW. This is critical in understanding the system's response to abrupt changes in power demand. Fig. 6 reveals that following the abrupt change at the offshore VSC terminal, the active power supplied by the offshore AC network quickly adapts to the new reference value of 16 MW. This rapid adaptation proves the proper tuning and effectiveness of the PI controller parameters. The ability to quickly stabilize after such an abrupt change is vital for maintaining the overall stability and reliability of the power system, ensuring the offshore AC network can meet varying power demands efficiently. Meanwhile, the reactive power within the offshore AC network remains consistently stable at the desired +4 MVAR. The inner PI current controller regulates the variation in the *d*-axis current at offshore VSC terminal, with an observed overshoot of 16 %. This overshoot indicates a temporary deviation of the current, but the system quickly stabilizes, underscoring the robustness of the control mechanisms in place. The variation in the three-phase currents aligns directly with the change in the active power reference at the offshore VSC terminal. This behavior is expected since the active power supplied is related to the current flowing through the network. Notably, no change is observed in the *q*-axis current at the offshore VSC terminal, indicating that the reactive power component remains unaffected by the change in active power demand.

As a result of the increased supplied active power, there is a corresponding rise in the DC voltage. Nevertheless, the DC voltage PI controller for the onshore VSC promptly reacts to this increase, swiftly restoring the DC voltage to its designated reference value of 70 kV. It results in a minor overshoot of approximately 0.72 % at the onshore VSC terminal. This quick correction demonstrates the controller's efficacy in maintaining DC voltage stability even during significant active power change. Furthermore, observing the variation in the *d*-axis current and three-phase currents at the onshore VSC terminal enables correlation with the change in the active power reference at the offshore VSC terminal. Additionally, it can be noted that the amount of active power delivered onshore closely matches the supplied active power from the offshore VSC terminal. It implies effective power transfer between the two AC networks. Additionally, since the reference reactive power at the onshore VSC terminal is set to 0 MVAR to achieve unity power factor operation, the *q*-axis current at the onshore VSC terminal is zero.

These findings underscore that despite a significant abrupt change in active power at the offshore VSC terminal, the system exhibits remarkable stability, with an observed overshoot of 16 % in the *d*-axis current at offshore VSC terminal, approximately 0.72 % overshoot in DC voltage at onshore VSC terminal and no impact on the *q*-axis current or reactive power.

### 5.2 Case 2

**Fig. 7:**
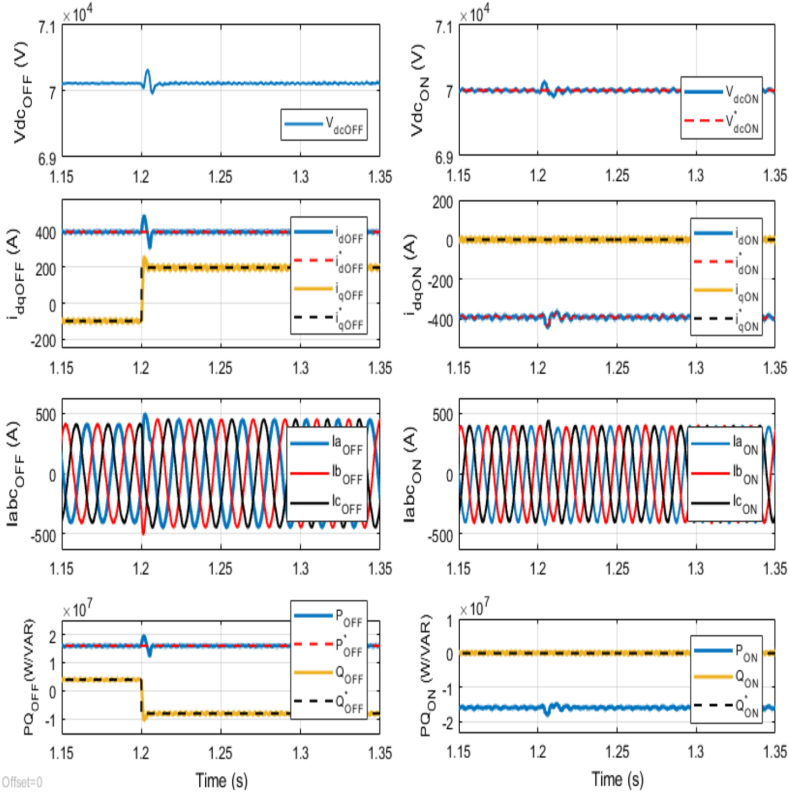
Dynamic performance of the VSC-based HVDC transmission system under abrupt change in Reference Reactive Power for Offshore VSC from +4 MVAR to −8 MVAR at time t = 1.2 s.

At 1.2 s, the reference reactive power is abruptly changed to −8 MVAR. Fig. 7 reveals a quick reactive power adjustment at the offshore VSC terminal to match the new reference, while the active power supplied by the offshore AC network remains constant at 16 MW. The overshoot of 18.5 % in the *q*-axis current, along with consequent variations in three-phase currents at the offshore VSC terminal, corresponds to the change in the reactive power reference. Moreover, a consequent variation in the *d*-axis current is also observed as a result of the abrupt change in the *q*-axis current at the offshore VSC terminal, attributed to the cross-coupling between the *d*-and *q*-axes currents. Conversely, at the onshore VSC terminal, where the reference reactive power is set to 0 MVAR for unity power factor operation, the *q*-axis current remains at zero. Notably, due to independent reactive power control at each terminal, changes in three-phase currents at the onshore VSC terminal are relatively minor compared to those at the offshore VSC terminal. Nonetheless, a slight variation occurs due to the small change in the *d*-axis current at the onshore VSC terminal. The DC voltage controller at the onshore VSC promptly responds to minor fluctuations in the measured DC voltage resulting from slight variations in the *d*-axis current. An observed overshoot of 0.4 % is noted in DC voltage at the onshore VSC terminal. Furthermore, it is crucial to highlight that the delivered active power closely matches that supplied from the offshore AC network. This demonstrates the high efficiency and reliability of power transmission between the two AC networks. Overall, these findings underscore that the system maintains stability despite a notable change in reactive power, with just an 18.5 % overshoot in the q-axis current at the offshore VSC terminal and only approximately 0.4 % overshoot in DC voltage at the onshore VSC terminal.

### 5.3 Case 3

**Fig. 8:**
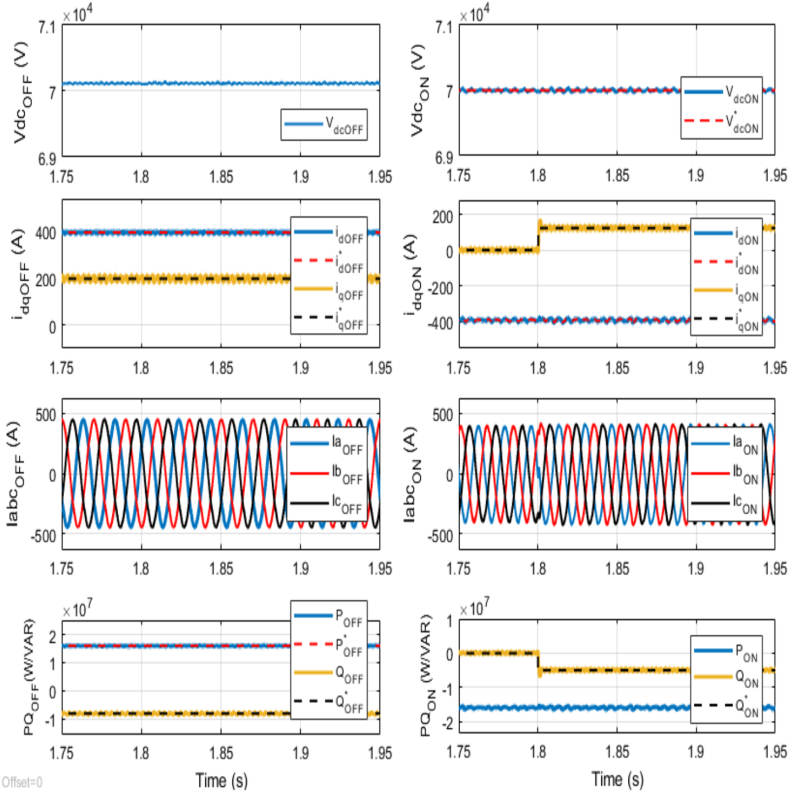
Dynamic performance of the VSC-based HVDC transmission system under abrupt change in Reference Reactive Power for Onshore VSC from 0 MVAR to −5 MVAR at time t = 1.8 s.

At 1.8 s, an abrupt change occurs in the reference reactive power alone at the onshore VSC terminal, with the new reference value set at −5 MVAR instead of 0 MVAR. Fig. 8 illustrates that at the onshore VSC terminal, the reactive power promptly aligns with this new reference setting, while no change occurs in the active power. The observed 19.2 % increase in the *q*-axis current at the onshore VSC terminal, accompanied by subsequent variations in three-phase currents, directly correlates with the change in reactive power reference. This dynamic response underscores the ability of the onshore VSC to promptly adapt to shifting grid conditions, ensuring stability and reliability in power delivery. Conversely, no variations are detected in the *d*-axis current and DC voltage at the onshore VSC terminal, highlighting the stability of these parameters despite the change in reactive power. It is noteworthy that the independent control of reactive power at each VSC terminal ensures that any fluctuations in three-phase currents at the offshore VSC terminal are negligible compared to those observed at the onshore VSC terminal. Furthermore, the close alignment between the active power delivered onshore and that supplied from the offshore terminal emphasizes the efficiency and reliability of power transmission between these interconnected systems. Thus, the rapid adjustment to the reactive power reference at the onshore VSC terminal illustrates the control capabilities in managing complex system dynamics.

### 5.4 Case 4

**Fig. 9:**
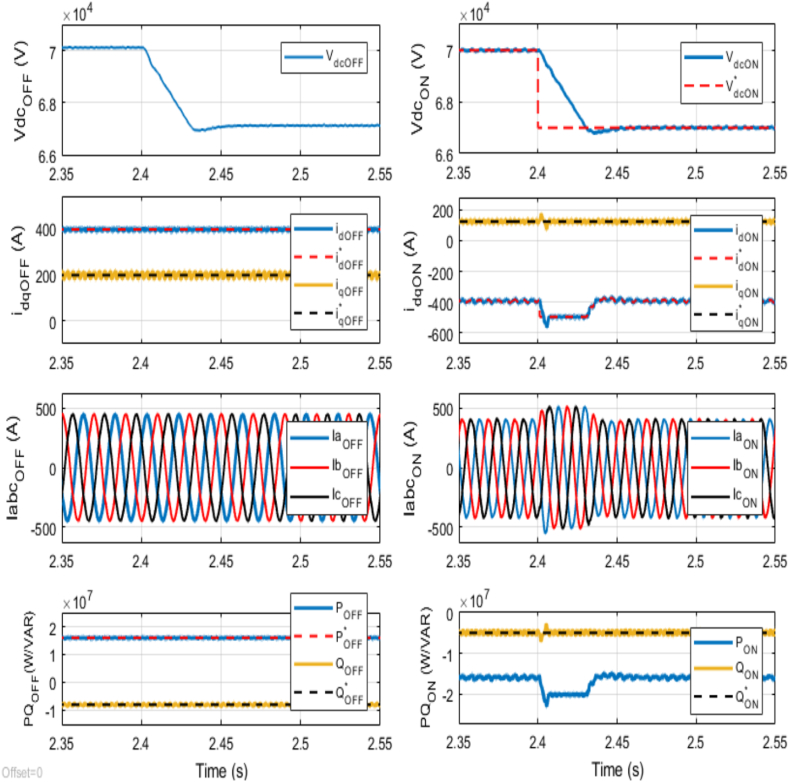
Dynamic performance of the VSC-based HVDC transmission system under abrupt change in Reference DC Voltage for Onshore VSC from 70 kV to 67 kV at time t = 2.4 s.

At 2.4 s, an abrupt change occurs in the reference DC voltage, dropping from 70 kV to 67 kV. As depicted in Fig. 9, the DC voltage PI controller for the onshore VSC quickly adjusts the measured DC voltage to align with this new reference. However, it slightly undershoots by 1.84 %, showcasing the PI controller's rapid but precise corrective action in managing DC voltage deviations. Concurrently, over at the offshore terminals, the measured active power is 16 MW, accompanied by a reactive power of −8 MVAR. The fluctuation in the *d*-axis current, and consequently, in the three-phase currents, as well as the active power at the onshore VSC terminal, directly correlates with the change in the measured DC voltage. Similarly, a corresponding variation in DC voltage is noticeable at the offshore VSC terminal.

Conversely, no changes are observed in the *q*-axis current or reactive power at the onshore VSC terminal, highlighting the system's stability in reactive power management despite the DC voltage adjustment. This stability is crucial for maintaining consistent grid operations and ensuring reliable power delivery. Moreover, during steady-state, the active power delivered onshore closely matches that supplied from the offshore AC network, underscoring the efficiency and reliability of power transmission. In contrast, there is no apparent variation in the *d-q* axes currents at the offshore terminal, resulting in no noticeable change in the three-phase currents at the offshore terminal following the change in DC voltage. In this manner, the system's capability to maintain stable DC voltage ensures a steady and smooth flow of power from the offshore to onshore AC networks.

These adjustments, executed within a remarkable 0.04 s timeframe, underscore the system's robustness and seamless transition to new reference settings, thereby ensuring stable and efficient power transfer in VSC-based HVDC transmission systems within practical applications.

## 6 Experimental validation in real-time

**Fig. 10:**
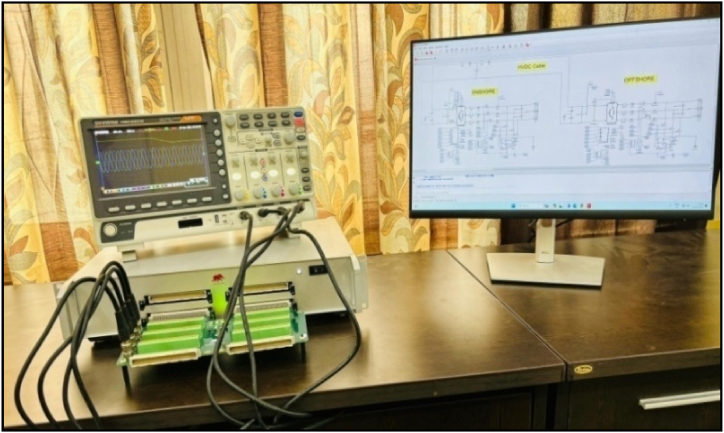
An experimental setup for real-time validation of control design in the VSC-based HVDC transmission system, employing the Typhoon HIL 604 simulator.

This section presents an experimental validation of the offline simulation results, assessing the dynamic response of the proposed VSC-based HVDC transmission system to abrupt changes in reference electrical parameters. These include active power and reactive power at the offshore VSC terminal, as well as reactive power and DC voltage at the onshore VSC terminal. The evaluations are conducted in real-time using the Typhoon HIL604 simulator, as depicted in Fig. 10.

**Fig. 11:**
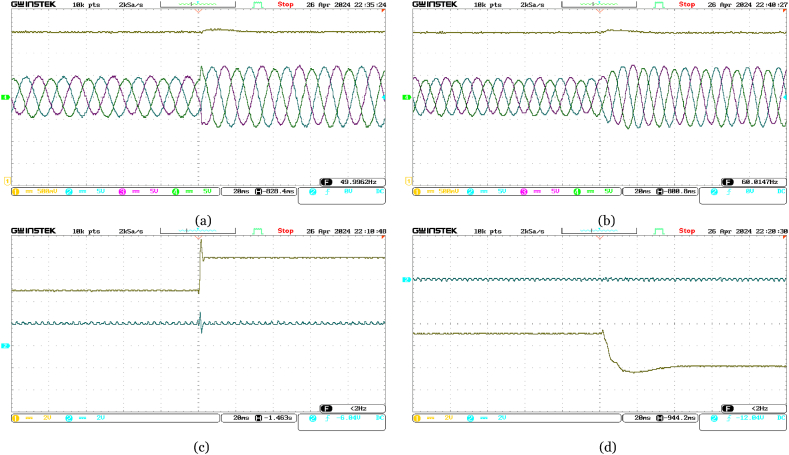
Experimental performance validation of the VSC-based HVDC system under abrupt change in Reference Active Power for Offshore VSC from 10 MW to 16 MW in real-time––(a) Ch. 1: DC voltage at the offshore VSC terminal (4 kV/div); Ch. 2–4: three-phase currents at the offshore VSC terminal (300 A/div); (b) Ch. 1: DC voltage at the onshore VSC terminal (4 kV/div); Ch. 2–4: three-phase currents at the onshore VSC terminal (300 A/div); (c) Ch. 1: active power supplied from offshore terminal (4 MW/div); Ch. 2: reactive power at offshore terminal (4 MVAR/div); (d) Ch. 1: active power delivered at onshore terminal (4 MW/div); Ch. 2: reactive power at onshore terminal (4 MVAR/div).

Initially set at 10 MW, the active power reference at the offshore VSC terminal abruptly changes to 16 MW. Real-time validation results, illustrated in Fig. 11, showcase the quick adaptation of the active power at offshore VSC terminal to meet the new reference, while maintaining stable reactive power. The increase in supplied active power leads to a rise in DC voltage, promptly regulated by the onshore DC voltage PI controller. Notably, variations in three-phase currents align closely with changes in active power.

**Fig. 12:**
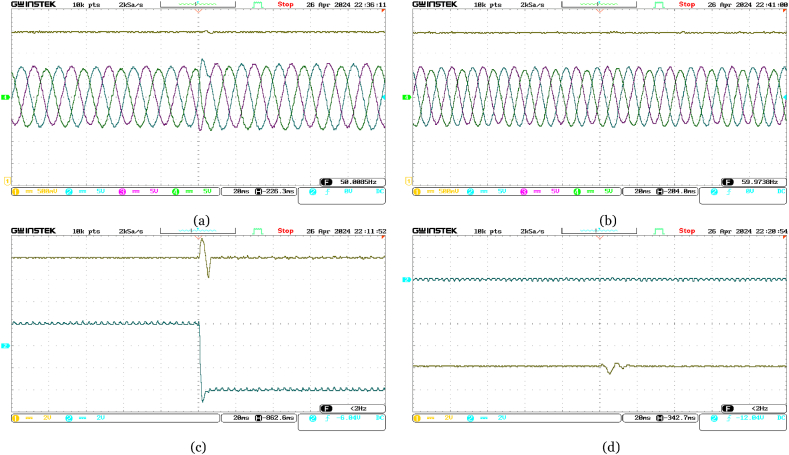
Experimental performance validation of the VSC-based HVDC system under abrupt change in Reference Reactive Power for Offshore VSC from +4 MVAR to −8 MVAR in real-time––(a) Ch. 1: DC voltage at the offshore VSC terminal (4 kV/div); Ch. 2–4: three-phase currents at the offshore VSC terminal (300 A/div); (b) Ch. 1: DC voltage at the onshore VSC terminal (4 kV/div); Ch. 2–4: three-phase currents at the onshore VSC terminal (300 A/div); (c) Ch. 1: active power supplied from offshore terminal (4 MW/div); Ch. 2: reactive power at offshore terminal (4 MVAR/div); (d) Ch. 1: active power delivered at onshore terminal (4 MW/div); Ch. 2: reactive power at onshore terminal (4 MVAR/div).

Moreover, as depicted in Fig. 12, when the reference reactive power changes abruptly from +4 MVAR to −8 MVAR, the reactive power at the offshore VSC terminal is promptly adjusted, while holding the active power from the offshore AC network constant at 16 MW. At the onshore VSC terminal, reactive power is observed to be around 0 MVAR to ensure unity power factor operation. Minor changes in three-phase currents occur as a result of changes in reactive power at each VSC terminal. The onshore DC voltage PI controller adeptly manages minor deviations in measured DC voltage.

**Fig. 13:**
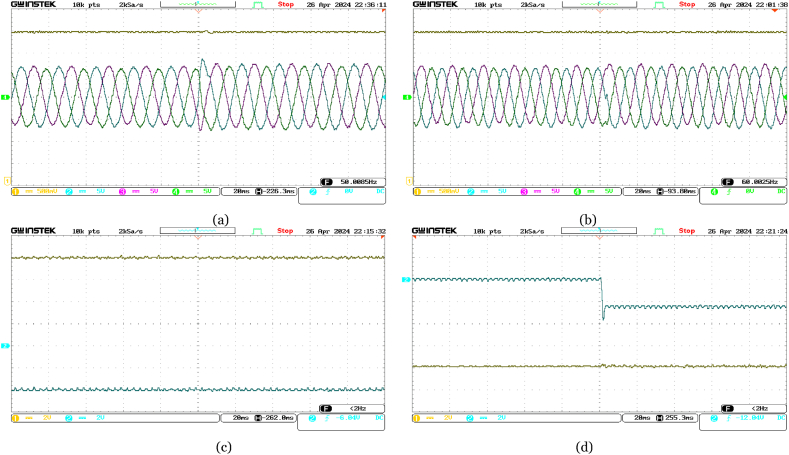
Experimental performance validation of the VSC-based HVDC system under abrupt change in Reference Reactive Power for Onshore VSC from 0 MVAR to −5 MVAR in real-time––(a) Ch. 1: DC voltage at the offshore VSC terminal (4 kV/div); Ch. 2–4: three-phase currents at the offshore VSC terminal (300 A/div); (b) Ch. 1: DC voltage at the onshore VSC terminal (4 kV/div); Ch. 2–4: three-phase currents at the onshore VSC terminal (300 A/div); (c) Ch. 1: active power supplied from offshore terminal (4 MW/div); Ch. 2: reactive power at offshore terminal (4 MVAR/div); (d) Ch. 1: active power delivered at onshore terminal (4 MW/div); Ch. 2: reactive power at onshore terminal (4 MVAR/div).

Furthermore, Fig. 13 highlights the responsive nature of the onshore VSC terminal when the reference reactive power changes abruptly from 0 MVAR to −5 MVAR. The onshore VSC quickly adjusts the reactive power accordingly, while the active power remains unchanged. The DC voltage at the onshore VSC terminal shows no significant variation. Due to independent reactive power control at each VSC terminal, variations in three-phase currents at the offshore VSC terminal are minimal compared to those at the onshore VSC terminal.

**Fig. 14:**
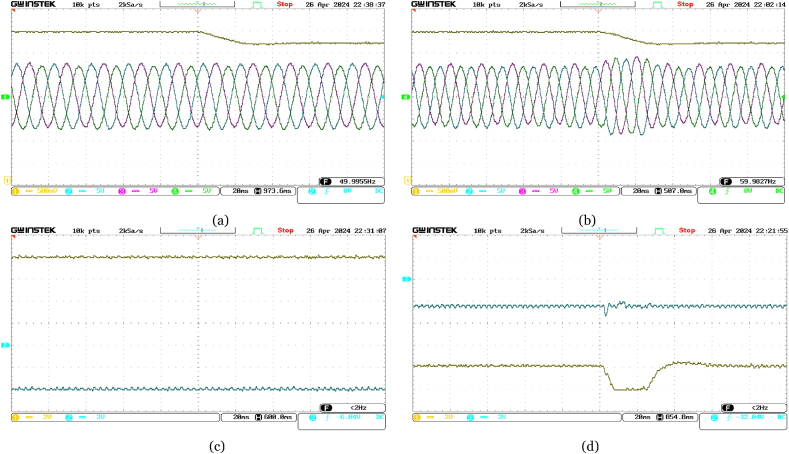
Experimental performance validation of the VSC-based HVDC system under abrupt change in Reference DC voltage for Onshore VSC from 70 kV to 67 kV in real-time––(a) Ch. 1: DC voltage at the offshore VSC terminal (4 kV/div); Ch. 2–4: three-phase currents at the offshore VSC terminal (300 A/div); (b) Ch. 1: DC voltage at the onshore VSC terminal (4 kV/div); Ch. 2–4: three-phase currents at the onshore VSC terminal (300 A/div); (c) Ch. 1: active power supplied from offshore terminal (4 MW/div); Ch. 2: reactive power at offshore terminal (4 MVAR/div); (d) Ch. 1: active power delivered at onshore terminal (4 MW/div); Ch. 2: reactive power at onshore terminal (4 MVAR/div).

Additionally, in Fig. 14, an abrupt drop in the reference DC voltage from 70 kV to 67 kV triggers quick adjustments by the DC voltage PI controller at the onshore VSC terminal. This ensures that the measured DC voltage converges to the new reference value with a slight undershoot. Consequently, a corresponding variation in DC voltage is observed at the offshore VSC terminal as well. Despite these changes, the measured active power remains constant at 16 MW with a reactive power of −8 MVAR at the offshore VSC terminal, while a transient fluctuation is observed in active power at the onshore VSC terminal. Remarkably, no change is observed in reactive power at the onshore VSC terminal. Besides, fluctuations in three-phase currents at the onshore VSC terminal directly correlate with changes in DC voltage, while negligible variations are observed at the offshore terminal.

Furthermore, the real-time validation results underscore the efficiency of power transfer between offshore and onshore AC networks. This efficiency is evidenced by the closely matched active power delivered onshore and the active power supplied from the offshore terminal during steady-state conditions in all test cases. Table 2 summarizes the observations from four test cases, affirming the efficacy of PI controller tuning and adaptability in promptly adjusting to new reference settings, ensuring stable power transfer, and validating the effectiveness of control strategies within the proposed VSC-based HVDC transmission system.

**Table 2:** Summary of observations across various test cases.

	OFFSHORE VSC TERMINAL				ONSHORE VSC TERMINAL			
Abrupt Changes →	$P_{OFF}^{*}$	$Q_{OFF}^{*}$	$Q_{ON}^{*}$	$V_{dcON}^{*}$	$P_{OFF}^{*}$	$Q_{OFF}^{*}$	$Q_{ON}^{*}$	$V_{dcON}^{*}$
	10 MW–16 MW	+4 MVAR to −8 MVAR	0 MVAR to −5 MVAR	70 kV–67 kV	10 MW–16 MW	+4 MVAR to −8 MVAR	0 MVAR to −5 MVAR	70 kV–67 kV
Parameters	Case–1	Case–2	Case–3	Case–4	Case–1	Case–2	Case–3	Case–4
DC voltage	*Consistent following an overshoot*	*Consistent following a minor overshoot*	*Consistent*	*Tracking with an undershoot*	*Consistent following an overshoot*	*Consistent following a minor overshoot*	*Consistent*	*Tracking with an undershoot*
*d-*Axis current	*Tracking with an overshoot*	*Consistent following an overshoot*	*Consistent*	*Consistent*	*Smooth Tracking*	*Consistent following a minor transient fluctuation*	*Consistent*	*Consistent after a transient variation*
*q-*Axis current	*Consistent*	*Tracking with an overshoot*	*Consistent*	*Consistent*	*Consistent*	*Consistent*	*Tracking with an overshoot*	*Consistent following a minor overshoot*
Three-phase current	*Apparent transient fluctuation*	*Apparent transient fluctuation*	*No Fluctuation*	*No fluctuation*	*No apparent transient fluctuation*	*Apparent transient fluctuation*	*Subtle yet apparent transient fluctuation*	*Steady following a transient variation.*
Active power	*Tracking with an overshoot*	*Consistent following an overshoot*	*Consistent*	*Consistent*	*Seamless tracking*	*Consistent following a minor transient fluctuation*	*Consistent*	*Consistent after a transient variation*
Reactive power	*Consistent*	*Tracking with an undershoot*	*Consistent*	*Consistent*	*Consistent*	*Consistent*	*Tracking with an undershoot*	*Consistent following a minor overshoot*

While offline simulations offer valuable insights into expected behavior, real-time validation ensures the robustness and reliability of the proposed control strategy in practical scenarios. This comprehensive approach ensured the proposed control strategy performed reliably under real-world conditions. By validating the consistency between results obtained from both methodologies, the study confidently demonstrated the reliability and effectiveness of the proposed control strategy across various test cases. This validation process not only strengthens the credibility of the findings but also underscores the practical relevance of the research in advancing control strategies for VSC-based HVDC transmission systems. Drawing from insights across diverse test cases, the research findings and their implications can be summarized as follows:

1. The research findings significantly impact real-world applications, particularly VSC-based HVDC transmission systems. The main findings of the case studies highlight substantial advancements in stability and dynamic performance achieved through optimized control strategies and finely-tuned PI controllers.
2. Specifically, the results demonstrate the exceptional adaptability of tuned PI controllers, quickly adjusting to new settings to ensure stable and efficient power transfer without severe transient fluctuations. This capability holds crucial implications for practical applications, enhancing the reliability and efficiency of power transmission across various operational environments.
3. Furthermore, the systematic approach to tuning PI controllers addresses a critical challenge often overlooked by designers. It improves closed-loop control performance while maintaining system robustness through the integration of finely-tuned PI controller parameters into the control system.
4. This approach is not limited solely to VSC-based HVDC transmission systems but can also be extended to other distribution system structures. It has the potential to provide a comprehensive solution for enhancing stability and dynamic response across various types of electrical grid configurations.

## 7 Conclusions

The authors propose the symmetric optimum tuning of PI controller parameters for a VSC-based HVDC transmission system. The effectiveness of these tuned PI parameters is demonstrated through four test cases, comprehensively evaluating the system's dynamic performance via both offline simulation and real-time experimental validation. The validation conducted through both offline simulations and real-time testing reinforces the practical applicability and reliability of the proposed control strategy. After considering all test cases, the following conclusions have been derived:

• Optimal control performance in VSC-based HVDC transmission relies on precise PI controller tuning.
• The symmetric optimum technique maximizes phase margin and minimizes closed-loop frequency response, relying solely on a few system parameters.
• The symmetric optimum technique, with a value of *a* = 2.4142, precisely tunes PI controller parameters for inner current control loops in offshore and onshore control systems, and the outer DC voltage loop in onshore control system, ensuring stable operation without oscillations.
• Stability and robustness are confirmed by a $45^{0}$ phase margin, along with a damping factor of 0.707 for complex conjugate poles in the closed-loop control system.
• The test cases validate the effectiveness of control strategies and the efficacy of tuned PI controllers, affirming the reliability of the proposed VSC-based HVDC transmission system for power transmission.
  o With an abrupt change in active power observed in Case 1 for offshore VSC, it is concluded that there is an adaptation to a new reference value of 16 MW. This adaptation is accompanied by a *d*-axis current overshoot of 16 % at the offshore VSC terminal. Notably, there is no change observed in the *q*-axis current, and consequently, no change in the reactive power at the offshore VSC terminal. Furthermore, the DC voltage remains at 70 kV, with only a minor 0.72 % overshoot observed at the onshore VSC terminal.
  oIn Case 2, there is a significant change in the reactive power for offshore VSC, which abruptly transitions to −8 MVAR while maintaining a steady active power of 16 MW. The adaptation to the new reference value of −8 MVAR results in a *q*-axis current overshoot of 18.5 % at the offshore VSC terminal. Despite this adjustment, the DC voltage is maintained at 70 kV, with just a 0.4 % overshoot observed at the onshore VSC terminal.
  oIn Case 3, there is a significant change in the reactive power for onshore VSC, which abruptly transitions to −5 MVAR while maintaining a steady DC voltage at 70 kV. The adaptation to the new reference value of −5 MVAR results in a q-axis current overshoot of 19.2 % at the onshore VSC terminal.
  oThe abrupt change in Case 4, where DC voltage drops from 70 kV to 67 kV, signifies a rapid adjustment to the new reference voltage of 67 kV, with a slight undershoot of 1.84 %. Notably, there is no change observed in the *d-q* axes currents, resulting in no changes in the active and reactive powers at the offshore VSC terminal.
• This way, the observation, coupled with dynamic performance analysis, provide invaluable insights into the effectiveness of tuned PI controllers for regulating active and reactive power, illustrating the system's remarkable adaptability as it promptly adjusts to new reference settings within 0.04s, thereby ensuring stable and efficient power transfer without experiencing severe transient fluctuations.

The work can be extended to advance control strategies for VSC-based HVDC systems, seamlessly integrating renewable sources, enhancing fault ride-through capabilities, and optimizing PI controller parameters within multi-terminal VSC-based HVDC networks. This extension may include comparative studies, such as evaluating the symmetric optimum PI tuning method against alternative approaches.

## Funding

Not Applicable.

## Data availability statement

Data included in article.

## CRediT authorship contribution statement

**Adesh Kumar Mishra:** Writing – original draft, Software, Formal analysis, Conceptualization. **Saurabh Mani Tripathi:** Investigation, Formal analysis, Conceptualization. **Omveer Singh:** Software, Formal analysis, Data curation. **Ankit Kumar Srivastava:** Investigation, Formal analysis, Conceptualization. **Thiyagarajan Venkatraman:** Writing – original draft, Visualization. **Raghavendra Rajan Vijayaraghavan:** Supervision, Formal analysis. **Sachin Kumar:** Visualization, Formal analysis, Data curation. **Rajvikram Madurai Elavarasan:** Writing – review & editing, Methodology, Formal analysis. **Lucian Mihet-Popa:** Writing – review & editing, Supervision, Formal analysis.

## Declaration of competing interest

The authors declare that they have no known competing financial interests or personal relationships that could have appeared to influence the work reported in this paper.

## Appendix A

**Parameters:** Rated HVDC system capacity: 20 MVA; Reference onshore DC voltage: 70 kV; Line-line voltage (offshore/onshore): 33 kV (RMS); Operating frequency (offshore): 50 Hz; Operating frequency (onshore): 60 Hz; Coupled AC reactors (offshore/onshore): 68 mH/0.06 Ω; DC capacitors ($C_{OFF}$): 400 μF; DC capacitors ($C_{ON}$): 325 μF; PWM switching frequency: 5 kHz; Inner loop sampling: 100 μs; Outer loop sampling: 1000 μs.
